# Exploring the heterogeneity of neural social indices for genetically distinct etiologies of autism

**DOI:** 10.1186/s11689-017-9199-4

**Published:** 2017-05-26

**Authors:** Caitlin M. Hudac, Holly A. F. Stessman, Trent D. DesChamps, Anna Kresse, Susan Faja, Emily Neuhaus, Sara Jane Webb, Evan E. Eichler, Raphael A. Bernier

**Affiliations:** 1 Department of Psychiatry and Behavioral Sciences, University of Washington, CHDD Box 357920, Seattle, WA 98195 USA; 2 Department of Genome Sciences, University of Washington School of Medicine, Seattle, WA 98195 USA; 3 Center for Child Health, Behavior, and Disabilities, Seattle Children’s Research Institute, Seattle, WA 98145 USA; 4 Boston Children’s Hospital and Division of Developmental Medicine, Harvard School of Medicine, Boston, MA 02215 USA; 5 Howard Hughes Medical Institute, Seattle, WA 98195 USA

**Keywords:** Autism spectrum disorders (ASD), Likely gene-disrupting mutations, Electroencephalography (EEG), Social cognition, Mu rhythm attenuation, Social perception, Molecular subtyping

## Abstract

**Background:**

Autism spectrum disorder (ASD) is a genetically and phenotypically heterogeneous disorder. Promising initiatives utilizing interdisciplinary characterization of ASD suggest phenotypic subtypes related to specific likely gene-disrupting mutations (LGDMs). However, the role of functionally associated LGDMs in the neural social phenotype is unknown.

**Methods:**

In this study of 26 children with ASD (*n* = 13 with an LGDM) and 13 control children, we characterized patterns of mu attenuation and habituation as children watched videos containing social and nonsocial motions during electroencephalography acquisition.

**Results:**

Diagnostic comparisons were consistent with prior work suggesting aberrant mu attenuation in ASD within the upper mu band (10–12 Hz), but typical patterns within the lower mu band (8–10 Hz). Preliminary exploration indicated distinct social sensitization patterns (i.e., increasing mu attenuation for social motion) for children with an LGDM that is primarily expressed during embryonic development. In contrast, children with an LGDM primarily expressed post-embryonic development exhibited stable typical patterns of lower mu attenuation. Neural social indices were associated with social responsiveness, but not cognition.

**Conclusions:**

These findings suggest unique neurophysiological profiles for certain genetic etiologies of ASD, further clarifying possible genetic functional subtypes of ASD and providing insight into mechanisms for targeted treatment approaches.

**Electronic supplementary material:**

The online version of this article (doi:10.1186/s11689-017-9199-4) contains supplementary material, which is available to authorized users.

## Background

The significant etiologic and phenotypic heterogeneity of autism spectrum disorder (ASD) [1] has made it challenging to target underlying mechanisms of ASD pathology. Considering that more than 1000 genes have been implicated in ASD [1, 2], recent initiatives have targeted genetic pathways [3, 4] and rare de novo likely gene-disrupting mutations (LGDMs) [5]. As such, a burgeoning “genetics-first” approach has been proposed to improve identification and characterization of genetic subtypes of individuals with ASD [6]. For instance, genetics-first studies have identified phenotypically distinct subtypes of autism for *CHD8* [7] and *DYRK1A* [8, 9] based upon behavioral and physical features within both children and animal models. However, the relevant contribution of genetic risk to aspects of the ASD phenotype (i.e., social communicative impairments) is poorly understood, especially for low-functioning individuals with ASD.

Recent work supports social perception as a possible neural index related to the hallmark social deficits in ASD [10–15]. Although the neural indices have been targeted in relation to copy number variations, such as the 16p11.2 locus [16, 17], little is known about neural patterns associated with LGDMs, likely due to the wide range of variability of specific gene expression across LGDMs. Our objective was to examine patterns of neural heterogeneity associated with LGDMs by completing a series of diagnostic and genetics-guided analyses of social perception. We hypothesized a diagnostic approach would indicate atypical social perception in ASD, consistent with theories of social brain dysfunction in ASD [15]. Then, as a preliminary exploration, we predicted that the heterogeneity associated with LGDMs would indicate potentially divergent patterns of social perception based upon LGDM function. Following work suggesting distinct functional roles for genes strongly expressed during embryonic development [5, 18], we tested children with and without a LGDM associated with embryonic development as a possible functional neurodevelopment pathway that contributes to a shared phenotype. There is a growing body of evidence suggesting early embryonic disruptions may be related to impairments in social behavior (e.g., lack of interest in conspecific proximity) [19, 20] and/or dysfunctional information encoding (i.e., intellectual or developmental delays) [21, 22]*.* The current study sought to add to this literature by addressing whether individuals with an embryonically expressed LGDM exhibit dysfunctional information habituation within the social domain.

We opted to target mu attenuation, which is specifically sensitive to detecting the movements associated with biological motion and is known as a reliable index of social perception in typical populations [23, 24]. Mu rhythm is typically defined as neural activity oscillations within the 8–12-Hz frequency range of electroencephalography (EEG) across electrodes above the sensorimotor cortex. During the observation and execution of biological motion, the underlying neural assemblies of the mu rhythm desynchronize [25, 26]. This desynchronization results in the reduction of oscillatory power (i.e., *attenuation* of the signal), with a greater reduction for conditions with social significance (i.e., biological motion relative to nonbiological motion) in children and adults; for a review, see [27].

In ASD, several studies suggest atypical mu attenuation in ASD (e.g., no discrimination for social relative to nonsocial observed motion) [12, 28–30] while other studies indicate no difference in ASD compared to typical controls [31–33]. Recent work by Dumas and colleagues [34] suggests that this discrepancy may be driven by the functional significance of the lower and upper mu rhythm bands. Notably, there is evidence that the lower mu rhythm (8–10 Hz) is more responsive to observed motion than the upper mu rhythm (10–12 Hz) [35], which may be indicative of bottom-up sensory processing [36, 37]. Previous studies also implicate that upper mu (or alpha) is more sensitive to top-down cognitive processing, such as self-monitoring within social contexts [38] or increasing cognitive demands [39, 40].

It is also possible that conflicting mu attenuation results reflect the underlying heterogeneity in ASD, potentially driven by genetic etiology. For instance, both disrupted social cognition and information habituation are associated with embryonically expressed LGDMs (e.g., *ADNP* [21], *POGZ* [22]). Yet, it is unclear whether social information habituation is also disrupted and the extent to which this profile is unique to children with an embryonically expressed LGDM. To date, only one study has tested the rate at which mu attenuation is modulated (i.e., habituates, sensitizes) in ASD [17]. In that study, children with ASD and an ASD-associated deletion or duplication at the *16p11.2* locus demonstrated divergent dynamic patterns of mu attenuation providing additional insight into the relationship between ASD-associated copy number variations (CNVs) and social neural phenotypes.

This study sought to characterize social motion discrimination in ASD within the upper and lower mu bands continuously over time to capture dynamic neural social indices that may be associated with LGDMs expressed in embryonic development. We tested mu attenuation and habituation first via diagnostic comparisons between typically developing (TYP) and ASD children and, second, via genetics-guided comparisons between children with and without LGDMs expressed preferentially in embryonic development (LGDM E+ vs. LGDM E−). Based upon prior work [12, 28–30], we predicted a lack of social motion discrimination in ASD relative to TYP and anticipated no habituation to either condition in ASD, consistent with [17]. We predicted that children with an embryonically expressed ASD-associated LGDM might have a more severely impacted social profile relative to LGDM E−, in part due to embryonic development as a (more) critical period for regulation of gene expression in support of brain development [41]. Lastly, we evaluated relationships between the neural social indices and individual predictors of social and cognitive behavioral features to better assess the specificity of mu attenuation to capture social processing.

## Methods

### Participants and clinical procedures

Thirty-nine children age 6–19 years participated in this study (see Table 1 for full characterization details). ASD-LGDM children (*n* = 13) were recruited to enroll in this study following participation in the Simons Simplex Collection or following independent genetic screening that identified a de novo ASD-associated LGDM with family-based exome sequencing studies [5] or companion molecular inversion probe-based (MIP) targeted resequencing of potential ASD loci [42, 43]. Post hoc clustering, based upon the functional role of the LGDM, tested genetically guided patterns of neural heterogeneity. Per Iossifov and colleagues [5, 18], LGDMs consisted of five genes primarily expressed in embryonic development (*ADNP* [44], *DYRK1A*, *n = 3* [8], *MED13L* [5]*, SETBP1* [45], and *SETD2*, *n* = 2 [46]) and four genes primarily expressed post-embryonic development (*CHD8*, *n* = 2 [7], *DSCAM* [42], *GRIN2B* [47], and *SCN2A* [48]). Comparison cases included equal number of age- and gender-matched children with idiopathic ASD (ASD-NON) and typical development (TYP). ASD-NON and TYP children were recruited from individuals who had previously completed other research projects within the research laboratory. None of the ASD-NON cases had an identified ASD-associated LGDM or otherwise specified ASD genetic events (e.g., ASD-associated copy number variation). TYP participants were defined as children without any parent-reported psychiatric or neurodevelopmental diagnoses and a lack of features of autism or subclinical communication concerns on the Social Responsiveness Scale-2 (SRS-2; i.e., all TYP participants scored under a *T*-score of 60) [49]. There were no differences in SRS-2 scores for ASD-LGDM or ASD-NON, *F*(1,24) = 0.008, *p* = .93. All research procedures conformed to regulations in accordance with the local ethical review board. Written informed consent was obtained from each parental representative(s). All children verbally assented to participate in the procedures, and written assent was obtained from children with a mental age of 7 or greater.

**Table 1:** Participant characterization

		Age	VIQ		NVIQ		SRS	
Group	*N* (*n* female)	*M* (SD)	*M* (SD)	Range	*M* (SD)	Range	*M* (SD)	Range
TYP	13 (3)	11.30 (3.79)	118.54 (12.07)	99–146	110.46 (7.91)	98–128	43.54 (3.73)	37–51
ASD-NON	13 (3)	10.26 (3.59)	104.62 (18.74)	66–134	109.54 (18.71)	85–138	74.62 (10.15)	62–90
ASD-LGDM	13 (3)	13.38 (2.92)	54.54 (32.45)	16–136	53.08 (30.42)	22–137	74.15 (16.31)	45–103
LGDM E+	7 (2)	8.23 (3.39)	51.83 (22.32)	24–84	48.67 (22.31)	22–86	81 (17.62)	55–103
LGDM E−	6 (1)	9.08 (3.10)	56.86 (40.96)	16–136	56.86 (37.41)	31–137	68.29 (13.65)	45–84

Participant characterization is provided for comparison groups (typical development, TYP; autism spectrum disorder nonrelated to a known genetic etiology, ASD-NON) and likely gene-disruptive mutations (LGDM), as well as the genetically guided clustering of LGDM with (E+) and without (E−) a primary role in embryonic development

*Abbreviations*: *VIQ* verbal intelligence quotient, *NVIQ* nonverbal intelligence quotient, *SRS-2* Social Responsiveness Scale-2

See Additional file 1: Table S1 for full clinical characterization of ASD participants. Diagnoses of autism were confirmed using the Autism Diagnostic Interview-Revised (ADI-R) [50, 51] and the Autism Diagnostic Observation Schedule-2 (ADOS-2) [49, 52]. Verbal and nonverbal IQ (VIQ, NVIQ) was assessed using the Wechsler Abbreviated Scale of Intelligence [10] or the Differential Ability Scales-Second Edition [50], depending on age. Due to the severe intellectual disabilities within the ASD-LGDM cases, IQ ratio scores (*n* = 3) were substituted when standard IQ deviation scores were not available. The ASD-LGDM cases were more cognitively impaired than both comparison groups in VIQ and NVIQ, *F*(1,24)’s > 23.21, *p*’s < .001. Thus, VIQ and NVIQ were included in the statistical models to account for known variation in IQ and explicitly tested as part of our third objective. We recognize that cognitive differences between LGDM and comparison groups (TYP, ASD-NON) is a limitation of our study; however, evidence suggests that mu attenuation during passive social perception is not linked to cognitive abilities [33]. In addition, the genetics-guided comparisons between LGDM E+ and LGDM E− were matched on age, IQ, autism severity (i.e., via the ADOS-2 score), and adaptive behavior (i.e., via the Vineland Adaptive Behavior Scales-2 [53]), *F*(1,11)’s < 2.15, *p* > .17.

### Identification of genetic variants

Small-molecule molecular inversion probes (smMIPs) [54] were designed to the coding portions of *CHD8*, *DSCAM*, *DYRK1A*, *GRIN2B*, *SCN2A*, *SETBP1*, *SETD2*, *ADNP*, and *MED13L* with a 5-bp single-molecule tag using a scoring algorithm described previously [55] in order to identify single-nucleotide variants (SNVs) and insertions/deletions (INDELs). Oligonucleotides (IDT, Coralville, IA) were ordered, and probes were pooled at an equal molar ratio and phosphorylated (1X pool). After initial testing, poor-performing smMIPs were repooled and phosphorylated in either a 10X or a 50X pool. A final working probe pool was created by combining the three pools so that the final concentration of each smMIP in the 10X and 50X initial pools was a 10- or 50-fold excess relative to the 1X pool. Genomic DNA capture, exonuclease treatment, and PCR amplification of each library were performed as previously described [42] with 120 ng of genomic DNA input. smMIP concentration was based on a ratio of 800 copies of each MIP to each haploid genome copy, based on the 1X pool concentration. We pooled barcoded libraries together and purified the pools with 0.8X AMPure XP beads (Beckman Coulter, Brea, CA) according to the manufacturer’s protocol. Pools were quantified in duplicate using the Qubit dsDNA HS Assay (Life Technologies, Grand Island, NY). All samples were sequenced on an Illumina MiSeq (Reagent Kit 300V2) or HiSeq 2000 according to the manufacturer’s instructions. Sequencing reads were analyzed using the mipgen analysis pipeline as described previously [55]. SNVs and INDELs were called using freebayes/0.9.14 and required a minimum of 8X coverage with a variant quality (QUAL) score greater than 20. Severe events (nonsense, frameshift, splice, and INDELs) were validated by Sanger sequencing.

In order to detect large CNVs, all samples (ASD-LGDM and ASD-NON) were run on custom genome-wide arrays (Agilent Technologies, Santa Clara, CA). Events for all ASD-LGDM samples have been previously published [56]. See Additional file 2: Table S2 for full genetic characterization of ASD participants.

### Social motion task

The objective of the social motion task was to examine the neural response to moving stimuli as it pertains specifically to social, biological agents more so than nonsocial, nonbiological objects. In the same paradigm and procedures as Hudac et al. [17], each child watched 12 total minutes of silent motion, alternating between conditions of social motion (i.e., hands clapping, animated person dancing), nonsocial motion (i.e., tubes swinging, animated ball bouncing), and no motion (i.e., the two empty backgrounds of motion videos). In this way, we can distinguish between the neural response for social and nonsocial motions, both relative to a baseline without motion. Each of the six 60-s videos was observed twice in one of two possible stimuli presentation orders. Between videos, participants were directed to take a break and the experimenter initiated the next video after confirmation that the participant was ready. Children were seated approximately 75 cm from a video monitor and were instructed to sit still and attend to the videos. Video stimuli were displayed using E-Prime 2.0 software (Psychology Software Tools, Inc., Pittsburgh, PA) at a size of 27 cm by 36.8 cm and subtended a visual angle of 20.4° by 27.6°.

### Electrophysiological recording

Continuous electroencephalogram (EEG) was recorded from a high-density 128-channel geodesic net using Net Station 4.3.1 software integrated with a 200-series high-impedance amplifier (Electrical Geodesics, Inc,, Eugene, OR). Electrode impedances were below 50 kΩ to maximize the signal-to-noise ratio, within the standard range for high-impedance amplifiers. During collection, EEG signals were referenced to the vertex electrode, analog filtered (0.1 Hz high-pass, 100 Hz elliptical low-pass), amplified, and digitized with a sampling rate of 500 Hz. A photocell recorded and marked the precise onset time of each video. During acquisition, researchers observed and marked periods containing movement and/or improper attention (e.g., participant looking away).

### Electrophysiological preprocessing

Methodological decisions were consistent with our previous study [17] and standard practices for processing EEG data [57]. Following data collection, continuous EEG was segmented into 2-s epochs starting with the onset of each 1-min video in order to generate 30 epochs per video. Epochs marked during acquisition as contaminated by movement or improper attention were removed. Automatic artifact detection rejected channels containing voltage shifts greater than 100 μV for each trial. In addition, trained research assistants reviewed and verified the automated artifact detection to ensure the data were sufficiently clean. If the channel was rejected for more than 50% of epochs (i.e., signifying poor data recording for that channel specifically), the channel was marked as a bad channel. After artifact detection, bad channels were corrected via interpolation from neighboring channels. Epochs were re-referenced to the average reference, excluding the rim channels due to the increased amount of noise from channels consistent with prior work (e.g., [58, 59]) in order to reduce the contribution of noise to the average reference.

Of the 120 possible epochs for each condition, all groups had more than 71.7% of artifact-free data for each condition (Table 2). Pairwise group comparisons indicated that both ASD groups had fewer artifact-free epochs for the nonsocial condition compared to TYP, but there were no differences between ASD-LGD and ASD-NON groups. There were no significant two-tailed Pearson correlations between the number of artifact-free epochs and cognitive predictors (VIQ, *r* = .31, *p* = .13; NVIQ, *r* = .25, *p* = .23) or ASD symptom severity (*r* = −.25, *p* = .22) for the ASD groups. In other words, the amount of data loss is fairly comparable across groups, and missing data due to artifact rejection is largely unrelated to the behavioral phenotype (i.e., missing at random). However, to ensure equal numbers of epochs were included from each group and individual, statistical analyses were restricted to the first 30 epochs for each condition with artifact-free data for each child.

**Table 2:** Artifact-free EEG data by group and condition

Mean (SD)	Social	Nonsocial
TYP	107.1 (21.4)	106.3 (14.5)
ASD-NON	98.8 (19.5)	92.1 (17.3)
ASD-LGDM	91.4 (28.5)	86 (24.8)
Group differences	Social	Nonsocial
TYP vs. ASD-LGDM	*t*(24) = −1.59, *p* = .13	***t*** **(24) =** −**2.55,** ***p*** **= .018**
TYP vs. ASD-NON	*t*(24) = 1.03, *p* = .32	***t*** **(24) = 2.27,** ***p*** **= .032**
ASD-NON vs. ASD-LGDM	*t*(24) = −.78, *p* = .44	*t*(24) = −.72, *p* = .48

Mean and standard deviations for the amount of acceptable, artifact-free epochs are presented for each group by condition. Results of independent-samples *t* tests are reported for pairwise comparisons between groups. Bold font highlights significant group differences.

*Abbreviations*: *TYP* typical development, *ASD* autism spectrum disorder, *NON* no known likely gene-disrupting mutation, *LGDM* likely gene-disrupting mutation

### Spectral analysis

To create neural indices of social perception, we computed power attenuation relative to the average baseline (no motion) for the first 30 artifact-free epochs in each condition (social, nonsocial) across central electrodes. Spectral power was calculated using fast Fourier transforms (FFTs) in MATLAB (version 7.12.0, R2011a; Natick, MA) on each 2-s epoch. Each power spectra was averaged across standard [12, 24] central electrodes clustered around the C3 (31, 32, 37, 38, 42, 43, 53, and 54) and C4 (80, 81, 87, 88, 94, 104, 105, and 106) positions. These electrodes are across to the sensorimotor region of the brain, thought to correspond to the mirror neuron system. Power attenuation was computed as the natural log of the ratio between the power of social motion or nonsocial motion epoch and the power of the individual’s average response during the no-motion condition. Subsequently, a value of zero represents no power attenuation relative to baseline [e.g., ln(condition/rest) = 0], and larger negative values represent greater power attenuation (i.e., social motion < rest). Only the first 30 artifact-free epochs in each condition (social, nonsocial) were included to ensure that each individual contributed an equal number of epochs. Power spectra included lower mu (8–10 Hz) and upper mu (10–12 Hz).

### Data analysis strategies

All analyses were conducted via SAS 9.3 (SAS Institute) using restricted maximum likelihood (REML) and Satterthwaite denominator degrees of freedom. A series of multilevel models were generated using PROC MIXED to describe the variances and covariances of attenuation, separately for each analysis and the two mu bands. All models included a random intercept for each individual.

As we were interested in dynamic changes in power attenuation varying by condition across exposure to the video (i.e., trial order), the final models included fixed effects for condition (0 = nonsocial), ASD diagnosis (diagnostic comparison model) or genetic group (genetics-guided comparison model), time (0 = trial 30, the last epoch), and subsequent interactions. Subject predictors were included as fixed effects to test additional contribution by each child’s age (0 = 12 years), FSIQ (0 = 100), VIQ (0 = 100), NVIQ (0 = 100), and gender (0 = male). None of the subject predictors significantly contributed to the models, *F*’s < 3.14, *p*’s > .086. However, all subject predictor fixed effects remained in the model to account for potential contributions to group variances.

## Results

By modeling dynamic changes over time (i.e., trial order), we were able to measure the rate by which mu attenuation habituates (becomes more positive over time) or sensitizes (becomes more negative over time). Specifically, neural indices were measured as the power attenuation difference between social and nonsocial conditions with the Tukey correction.

### Diagnostic comparisons (TYP and ASD)

We first examined mu attenuation related to social discrimination (social vs. nonsocial motion differences) using a diagnostic comparison of typical development and ASD. Full model results for each comparison are reported in Table 3.

**Table 3:** Diagnostic comparison MLM results

Effect	Lower mu	Upper mu
Condition	***F*** **(1,4635) = 27.88,** ***p*** **< .0001**	***F*** **(1,4635) = 9.43,** ***p*** **= .002**
Group	*F*(1,35) = 1.35, *p* = .254	*F*(1,34.9) = 0.66, *p* = .423
Condition by group	***F*** **(1,4635) = 4.75,** ***p*** **= .029**	***F*** **(1,4635) = 6.32,** ***p*** **= .012**
Slope	***F*** **(1,4635) = 19.64,** ***p*** **< .0001**	***F*** **(1,4635) = 11,** ***p*** **< .001**
Slope by condition	*F*(1,4635) = 3.21, *p* = .073	***F*** **(1,4635) = 3.86,** ***p*** **= .049**
Slope by group	***F*** **(1,4635) = 4.85,** ***p*** **= .028**	*F*(1,4635) = 2.43, *p* = .119
Slope by condition by group	*F*(1,4635) = 0.47, *p* = .495	*F*(1,4635) = 0.13, *p* = .717
VIQ	*F*(1,33) = 0.24, *p* = .631	*F*(1,33) = 1.83, *p* = .186
NVIQ	*F*(1,33) = 1.66, *p* = .207	*F*(1,33) = 1.92, *p* = .175
Age	*F*(1,33) = 1.16, *p* = .290	*F*(1,33) = 1.71, *p* = .200
Gender	*F*(1,33) = 2.07, *p* = .160	*F*(1,33) = 2.9, *p* = .098

Multilevel model results for diagnostic comparison by group (TYP vs. ASD) for lower mu (8–10 Hz) and upper mu (10–12 Hz) attenuation. Bold denotes significant effect

**Fig. 1:**
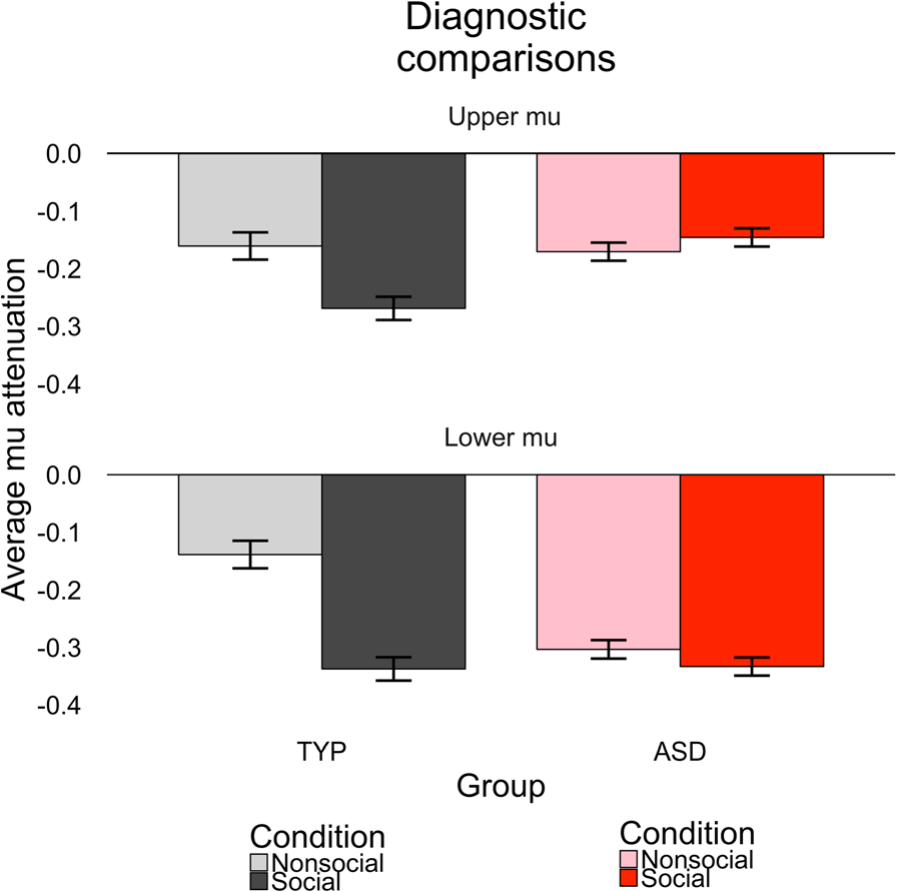
Diagnostic comparisons of overall mu attenuation between TYP and ASD. Power attenuation for social (*dark black*/*dark red*) and nonsocial (*light pink*/*light gray*) motions is averaged and plotted for typically developing children (*TYP*, *black*/*gray*) and children with ASD (*ASD*, *red*/*pink*). *Error bars* reflect 1 standard deviation

Average mu attenuation for each group is illustrated in Fig. 1. Omnibus tests (see Table 3) for both mu bands indicated main effects of condition, such that there was more mu attenuation for social relative to nonsocial motion. As expected, the TYP group exhibited social discrimination with greater social than nonsocial attenuation within both lower mu, *F*(1,4635) = 11.70, *p* = .0006, and upper mu, *F*(1,4635) = 20.87, *p* < .0001. In contrast, the ASD group only exhibited this pattern of social discrimination within lower mu, *F*(1,4635) = 7.22, *p* = .0073, and no discrimination within upper mu, *F*(1,4635) = 0.23, *p* = .63.

**Fig. 2:**
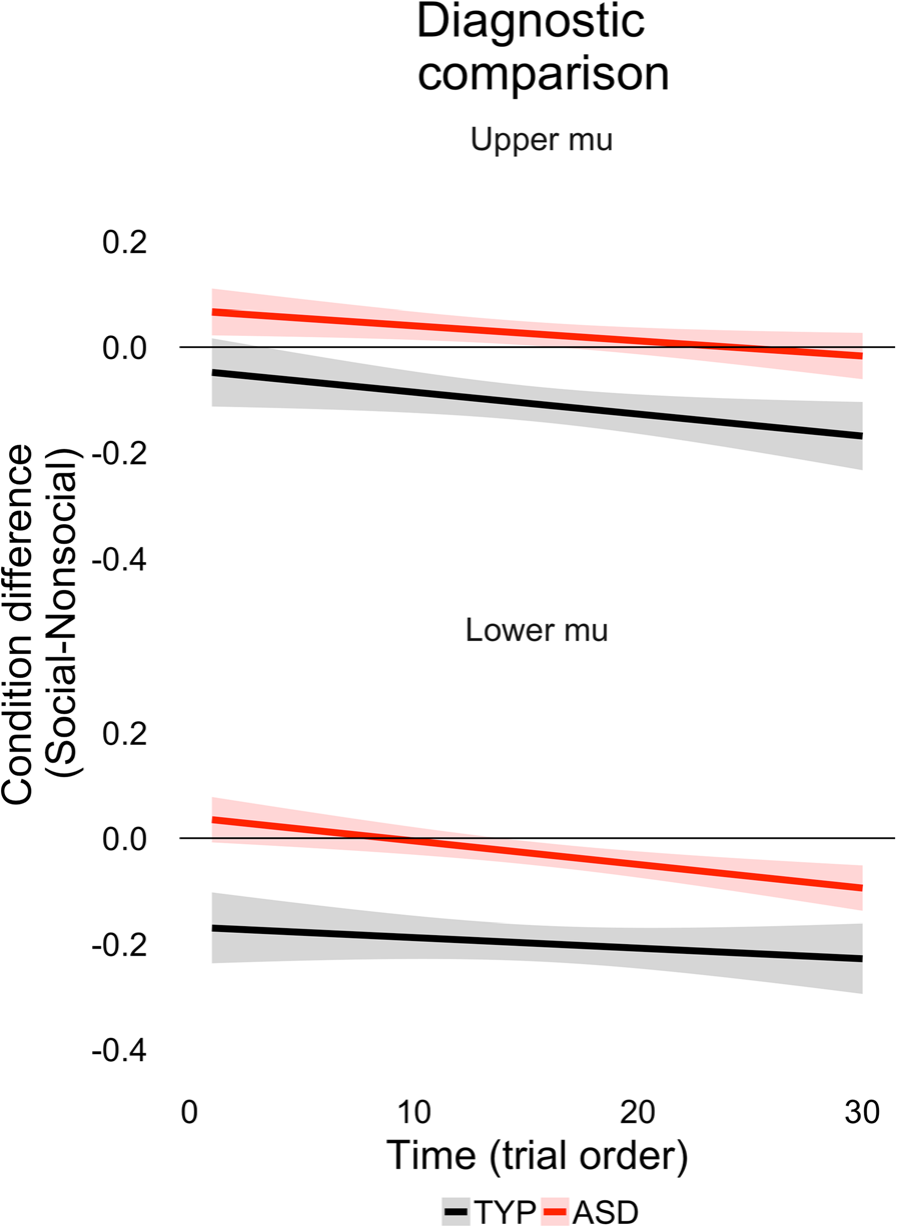
Diagnostic comparisons (TYP vs. ASD) of ongoing dynamic changes of mu attenuation between social and nonsocial motion perception. Power attenuation differential between conditions is averaged across subjects and plotted for TYP (*black*) and ASD (*red*). Positive values indicate more mu attenuation for nonsocial relative to social motion perception. Negative values indicate more mu attenuation for social relative to nonsocial motion perception. *Shading* reflects 80% confidence intervals

The omnibus tests also indicated a main effect of slope, such that mu attenuation habituated over the course of the experiment (i.e., collapsing across condition). Both groups exhibited different social and nonsocial slopes within lower mu [TYP: *F*(1,4635) = 19.21, *p* < .0001; ASD: *F*(1,4635) = 7.16, *p* = .0075], such that both groups habituated to nonsocial motion (TYP slope = .007; ASD slope = .004) more quickly than social motion (TYP slope = .005; ASD slope = 0). Within upper mu, only the TYP group had different dynamic patterns, [TYP: *F*(1,4635) = 11.11, *p* = .0009; ASD: *F*(1,4635) = 0.29, *p* =.59]. Neither group habituated or sensitized to social motion (slopes = 0), but only the TYP group habituated to nonsocial motion (TYP slope = .006, ASD slope = .003). Figure 2 illustrates the relative condition discrimination across trial order, highlighting in the ASD group both the increasing difference between social and nonsocial lower mu attenuation and the lack of discrimination in upper mu attenuation.

### Genetics-guided comparisons of the LGDM group (E− vs. E+)

Next, we sought to explore whether these patterns were consistent within a subsample of the ASD group with a known genetic etiology of ASD. In particular, we were interested in post hoc clustering comparisons based upon gene function given prior work targeting functional classes of LGDMs [5, 18]. To this extent, we evaluated mu attenuation related to social discrimination within the LGDM group to compare LGDMs associated with and without strong gene expression during embryonic development (LGDM E+ vs. LGDM E−).

**Fig. 3:**
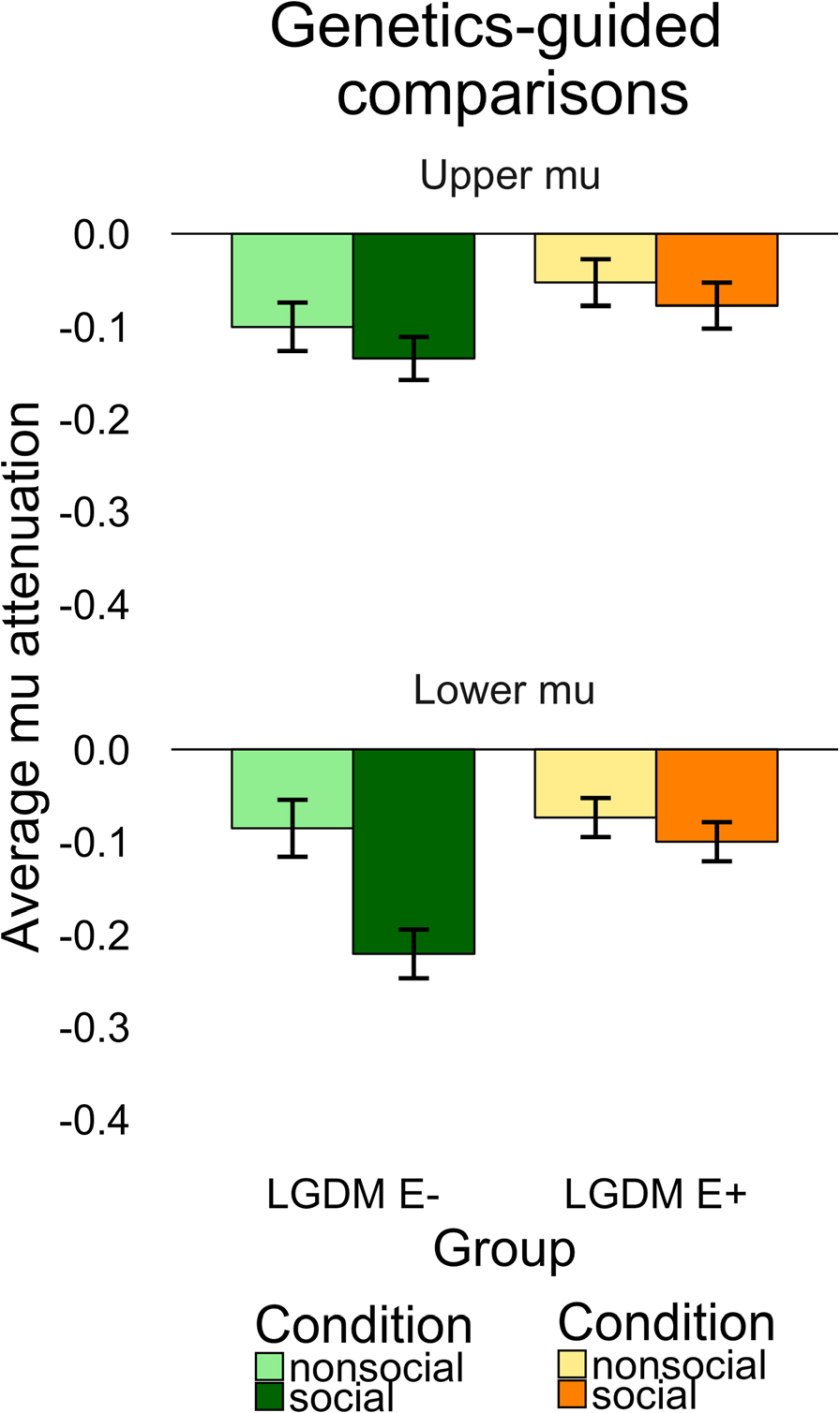
Genetics-guided comparisons of overall mu attenuation between LGDM E+ and LGDM E−. Power attenuation for social (*dark green*/*orange*) and nonsocial (*light green*/*yellow*) motions is averaged and plotted for children with an LGDM that is primarily expressed during embryonic development (*LGDM E+*, *orange*/*yellow*) and children with an LGDM that is not primarily expressed during embryonic development (*LGDM E−*, *light green*/*dark green*). *Error bars* reflect 1 standard deviation

**Fig. 4:**
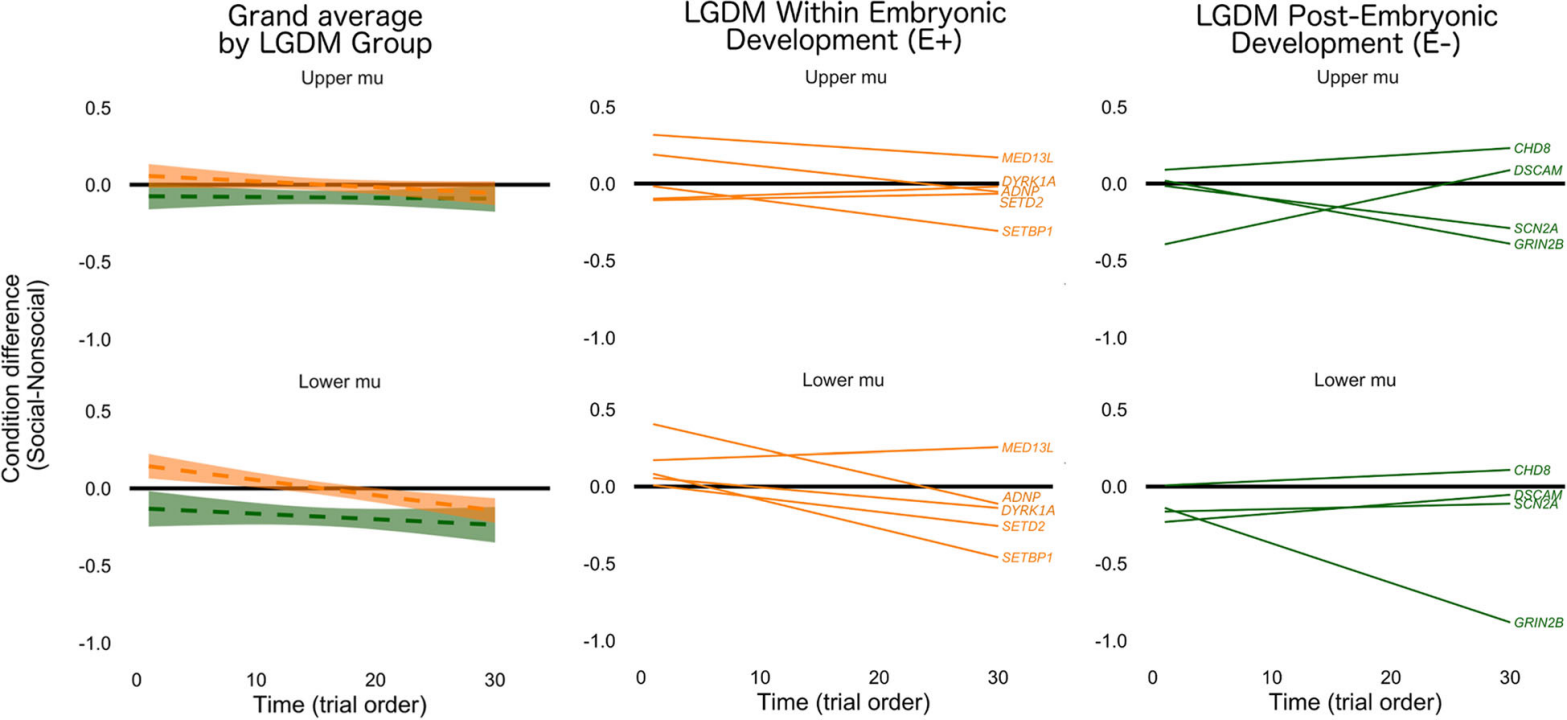
Genetics-guided comparisons (LGDM E+ vs. LGDM E−) of ongoing dynamic changes of mu attenuation between social and nonsocial motion perception. Power attenuation differential between conditions is averaged across subjects and plotted for LGDM E+ (*orange*) and LGDM E− (*green*). Positive values indicate more mu attenuation for nonsocial relative to social motion perception. Negative values indicate more mu attenuation for social relative to nonsocial motion perception. *Left panel*: group grand averaged values. *Shading* reflects 80% confidence intervals. *Middle panel*: LGDM primarily expressed within early embryonic development (LGDM E+). Note that individuals with shared LGDMs (i.e., DYRK1A, *n* = 3; SETD2, *n* = 2) are averaged into a single slope. *Right panel*: LGDM primarily expressed post-embryonic development (LGDM E−). Note that individuals with shared LGDMs (i.e., *CHD8*, *n* = 2) are averaged into a single slope

Full model results are reported in Table 4, and patterns of average mu attenuation and dynamic changes over trial order are illustrated in Figs. 3 and 4, respectively. Omnibus tests (see Table 4) indicated that there were no condition, group, or slope effects within upper mu; thus, we focus here on the lower mu band. A main effect of condition indicated that both LGDM E+ and LGDM E− groups exhibited social discrimination with greater social than nonsocial attenuation within lower mu.

**Table 4:** LGDM comparison MLM results

Effect	Lower mu	Upper mu
Condition	***F*** **(1,1541) = 12.18,** ***p*** **< .001**	*F*(1,1541) = 0.69, *p* = .406
Group	*F*(1,9.33) = 0.24, *p* = .634	*F*(1,7.79) = 0.03, *p* = .865
Condition by group	*F*(1,1541) = 0.01, *p* = .914	*F*(1,1541) = 0.06, *p* = .809
Slope	*F*(1,1541) = 0.01, *p* = .904	*F*(1,1541) = 0.52, *p* = .469
Slope by condition	***F*** **(1,1541) = 4.14,** ***p*** **= .042**	*F*(1,1541) = 0.04, *p* = .849
Slope by group	***F*** **(1,1541) = 4.11,** ***p*** **= .043**	*F*(1,1541) = 2.98, *p* = .084
Slope by condition by group	F(1,1541) = 1.55, *p* = .213	*F*(1,1541) = 0.16, *p* = .687
VIQ	*F*(1,7) = 0.71, *p* = .426	*F*(1,7) = 2.71, *p* = .144
NVIQ	*F*(1,7) = 1.6, *p* = .246	*F*(1,7) = 0.86, *p* = .385
Age	*F*(1,7) = 0.09, *p* = .776	*F*(1,7) = 0.08, *p* = .785
Gender	*F*(1,7) = 1.56, *p* = .252	*F*(1,7) = 4.41, *p* = .074

Multilevel model results for genetically guided comparison by group (LGDM E+ vs. LGDM E−) for lower mu (8–10 Hz) and upper mu (10–12 Hz) attenuation. Bold denotes significant effect

*Abbreviations*: *LGDM E+* likely gene-disrupting mutations primarily expressed during embryonic development, *LGDM E−* likely gene-disrupting mutations not primarily expressed during embryonic development

Omnibus tests (see Table 4) indicated slopes differed by condition and by group. Both groups exhibited different social and nonsocial slopes within lower mu [LGDM E−: *F*(1,1541) = 4.89, *p* = .027; LGDM E+: *F*(1,1541) = 7.52, *p* = .0062]. Neither group habituated or sensitized to nonsocial motion (slopes = 0), and the LGDM E− group also did not habituate or sensitize to social motion (slope = 0). However, the LGDM E+ group sensitized to social motion (slope = −.007), such that condition discrimination initially indicated more mu attenuation for nonsocial motion and ended with more mu attenuation for social motion at the end of the experiment. In other words, similar to typical development, both LGDM groups exhibited more social mu attenuation by the end of the experiment, but this effect was only shown after a delayed amount of time in the LGDM E+ group.

### Relationships between neural indices and individual differences

**Fig. 5:**
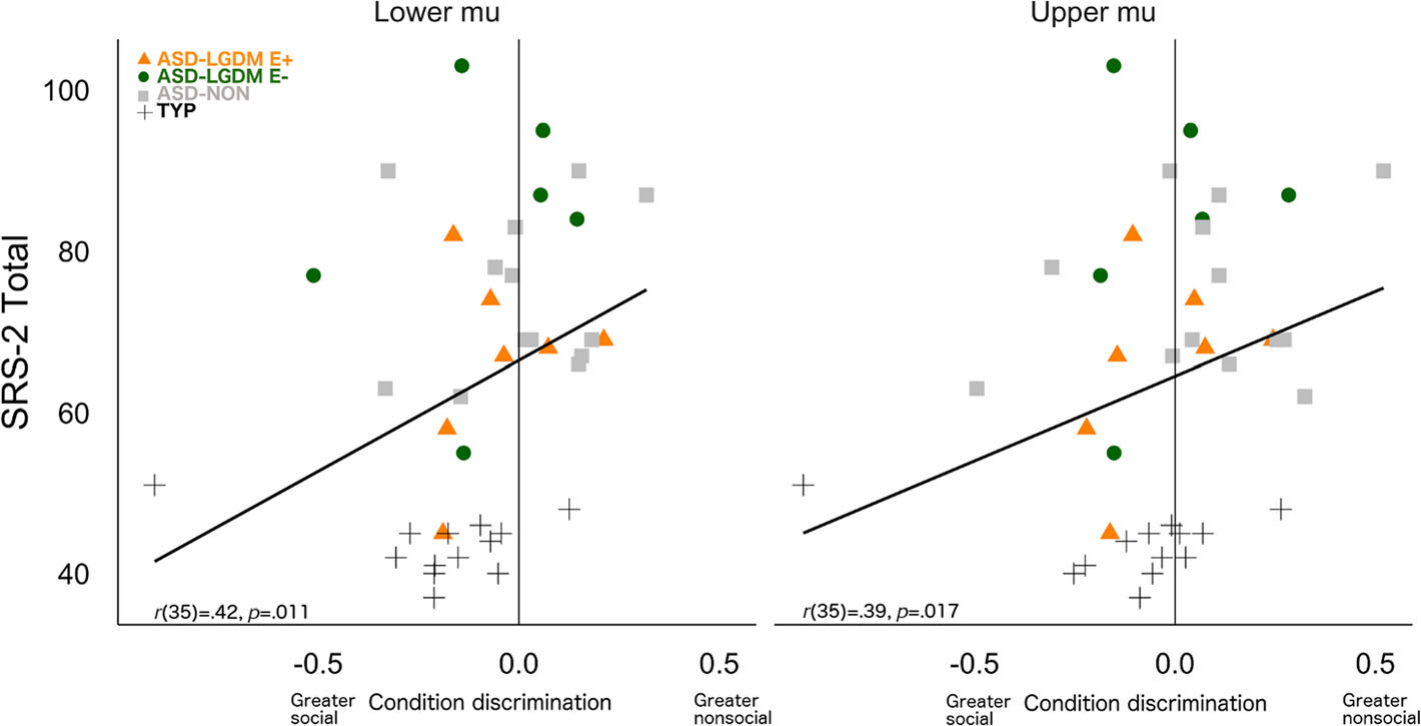
Neural social index correlations with social responsiveness (SRS-2 total score). Individual children are denoted by group: typical development (*TYP*, *black cross*), idiopathic ASD (*ASD-NON*, *gray box*), children with ASD and an LGDM associated with embryonic development (*ASD-LGDM E+*, *orange triangle*), and children with ASD and an LGDM not associated with embryonic development (*ASD-LGDM E−*, *green circle*)

Lastly, we wanted to determine the specificity of this measure of neural indices in relation to social and cognitive behavioral features for each child. Due to the known variability of nonclinical populations [60], we included all subjects in this analysis. We examined relationships to condition discrimination (i.e., the amount of difference between social and nonsocial mu attenuation). Partial correlation analyses (*p* < .05, controlling for age and NVIQ) indicated that condition discrimination was related to better overall social responsiveness (SRS-2) [49] for both lower mu, *r*(35) = .42, *p* = .011, and upper mu, *r*(35) = .39, *p* = .017 (Fig. 5). There were no significant associations between cognitive measures (VIQ, NVIQ) and condition discrimination within lower or upper mu as tested by Pearson correlations, *p*’s > .15.

## Discussion

Social impairments are a hallmark of ASD, yet phenotypic and genetic heterogeneity is thought to contribute to discrepant evidence in the literature. We explore a neural mechanism associated with ASD by considering patterns of social discrimination as measured by mu attenuation over time for children with different genetic etiologies. From the diagnostic comparisons, we show aberrant patterns of mu attenuation in ASD are specific to the upper mu band, while the lower mu band reflects less atypical patterns, consistent with prior work [34]. The dynamic patterns indicate that children with ASD show an increasing lower mu difference between social and nonsocial motions, which may help resolve diagnostic inconsistencies within the literature. For instance, prior evidence of atypical mu attenuation in ASD between observed motion conditions (i.e., social relative to nonsocial motion, as in the current study) [12, 28–30] has relied on individual averages. Our findings suggest that the discrimination pattern may not be evident if there are too few trials (i.e., before children with ASD habituated to nonsocial motion observations). Although it is a concern that children with ASD did contribute fewer trials than the typical controls, the eventual condition differentiation in ASD (i.e., noted by approximately trial 15 in Fig. 2) indicates that our study had a sufficient number of trials.

Mu attenuation has been proposed to reflect a human corollary to the mirror neuron system [24, 61], which describes activation recorded over the sensorimotor cortex during both action execution and observation of human actions. Although it is possible that mu attenuation reflects the conductance of occipital or posterior alpha rhythm more broadly (i.e., responsivity to general motion information) [62], our results indicate differentiation of social and nonsocial motions. One working hypothesis of ASD suggests mirror neuron system deficits that disrupt neural correlates supporting the action/observation system, subsequently eliciting atypical mu attenuation [63]. Aligned with this theory, other evidence suggest that atypical functioning of the mirror neuron system may lead to a downstream effect of poor imitative abilities [33] or disrupted higher order social cognitive abilities (i.e., theory of mind) [64]. However, it is important to note that similar to prior work by Dumas and colleagues, we found mu attenuation diagnostic differences within the upper mu band (10–12 Hz), but no group difference within the lower mu band (8–10 Hz). This is consistent with prior work suggesting that this lower frequency may reflect primary sensory processing [36, 37] that habituates over the course of the exposure. Yet, sensory processing of biological motion occurred more rapidly in the TYP group compared to longer processing in the ASD group, perhaps indicative of functional connectivity reductions related to social cognition [65]. Our results offer further evidence of atypical mu attenuation patterns in ASD, although unique neural mechanisms underlying atypical social discrimination may be derived from specific genetic etiologies. In other words, a mirror neuron hypothesis may indeed describe a subset of children with ASD, while a more general, distributed network of neural correlates may be impacted in other ASD subgroups.

As part of a preliminary analysis, we examined the neural social indices associated with different functional genetic roles of LGDMs as a first step to explore a possible shared neural social phenotype. We implemented a post hoc clustering strategy in order to examine potential convergent pathways between LGDMs that are and are not functionally expressed during embryonic development [18, 66]. The choice to cluster LGDMs around functional expression during embryonic development is based on early genetic regulatory control supporting regional differentiation within the embryonic brain [67, 68], including key social neural structures (e.g., amygdala). We had predicted that the LGDM within the embryonic development group would have a more severely disrupted neural social index due to evidence from animal and human models indicating significant impairments related to social behavior [19, 20] and/or information encoding [21, 22]. The results indicated that children with an LGDM primarily expressed during embryonic development exhibit sensitization of lower mu attenuation to social motion. In other words, these children initially exhibited more mu attenuation for nonsocial motion, but eventually demonstrate more for social motion. This pattern was distinct from children with an LGDM not primarily expressed during embryonic development that exhibited greater lower mu attenuation discrimination throughout the entire experiment (i.e., greater mu attenuation to social than nonsocial motion beginning at the first few trials).

Our results suggest that social motion perception may be conserved despite early genetic disruption, though the delayed processing supports the notion of potentially delayed information processing. It is important to note that this delay was specific to the social motion condition (increasing neural response over time) but not the nonsocial motion condition (i.e., no change over time), which may help clarify the mechanism by which prior models [19, 20] derive impaired social behavior. An interpretation of the results may be that children with an LGDM primarily expressed during embryonic development are increasing their attention to, or interest in, social stimuli after an initial period, which may reflect a delayed social engagement (e.g., motivation or salience). One explanation may be that the impact of embryonic genes on social perception is greater [69], suggesting that functional timing of genetic expression may differentially affect the neural social phenotype. Importantly, these findings align with genetics research indicating that ASD genes converge on several select pathways [70, 71], which may help to further explain the underlying neural social heterogeneity.

An important limitation of the current study is the continued genetic heterogeneity despite functionally classifying the expression of LGDM within early development. Within our LGDM groups, there are only several children with a shared LGDM (i.e., *SETD2*, *n* = 2; *DYRK1A*, *n* = 2; *CHD8*, *n* = 2). Thus, the discoveries of this work are not to be taken as firm conclusions, but rather considered in order to motivate and guide continued use of a genetics-first approach to elucidate potential etiological mechanisms of ASD. For instance, most of the children within the early embryonic LGDM group exhibit the social sensitization pattern described here (six out of eight cases; see Additional file 3: Figure S1 for individual patterns), except for one child with *MED13L* and one child with *DYRK1A*. In part, this qualitative finding is consistent with the overall group clustering approach indicating delayed social processing, suggesting a potential neural index associated with this particular genetic etiology. However, the specificity for specific LGDMs may be poor, considering that only two out of three children with a *DYRK1A* LGDM exhibited this pattern. Similar to prior work linking core social symptoms to biomarkers of ASD [11, 72–76], we encourage the use of this data as a way to bridge the gap between genetic and phenotypic characterization as a means to facilitate the discovery of ASD etiological mechanisms and accelerate progress for ASD therapeutic interventions.

It may be surprising that our task elicited mu attenuation during nonsocial motion observation (i.e., ball bouncing, tubes swinging) that is not biological and subsequently should not be simulated within the action/observation system. However, to a large extent, the majority of studies implementing mu attenuation as an outcome utilized comparisons between self-executed, social observed, and nonsocial observed motion. It may be the case that by engaging the motor execution system during these tasks, the threshold for the action/observation system is elevated, reducing the amount of mu attenuation for nonsocial comparisons. In fact, neural regions implicated in mu suppression during execution vs. observation [77] involve regions that also play a role in general motion perception, including the occipital, premotor, and somatosensory cortices. Moreover, this study replicated prior work with this same task that indicated a modest degree of mu attenuation to nonsocial motion, in addition to social motion [17]. We posit that our task measured more globally distributed neural differences between social and nonsocial motions compared to other tasks that have used self-initiated actions to target the premotor cortex. Of note, this passive viewing task is more conducive for children with reduced capacity for following behavioral instructions (i.e., to make self-initiated motions), while still providing a robust neural index, which specifies individual patterns.

The neural social indices were correlated with features of social cognition (i.e., social responsiveness), particularly with the lower mu band. This finding is compelling evidence that these indices accurately capture subtle levels of social impairments in vivo, as opposed to relying on parental reports (e.g., SRS-2). Additionally, average patterns of mu attenuation were unaffected by general cognition, despite drastic cognitive differences for children with a LGDM. Although this may not negate a contributory role of cognitive ability for higher-order operations related to social motion (e.g., action prediction), this evidence from this study suggests that motion perception is intact for children with lower cognitive abilities (i.e., cognitive scores under 50). Much of the existing research investigating neural social indices is restricted to children and adults with moderate to average cognitive capabilities. The majority of ASD-LGDM cases with low verbal IQ show typical mu attenuation patterns (i.e., greater for social motion in five out of eight cases with verbal IQ < 50). Taken together, these neural social indices can provide a robust characterization of the underlying neural mechanisms supporting social cognition, regardless of level of cognitive function, thereby improving our understanding of the social phenotype.

This study is the first to use a genetics-first approach to explore the genetic etiologies of autism associated with severe LGDMs in the context of neural social indices. Our use of a unique statistical method to measure ongoing dynamic changes associated with social motion perception demonstrates the utility of this method to better understand underlying processes relevant to ASD and LGDMs. Although this study is limited by a small sample size and thus should be considered exploratory, the analysis of neural social phenotypes based on functional clustering offers a promising approach for narrowing in on convergent pathways that may reflect shared phenotypes and provide insight for targeted treatment [5, 18]. Future research will need to take into account the variety and combination of genetic functional roles. Ongoing efforts to recruit a larger, more genetically homogenous group will help target specific functional outcomes during early childhood and adolescence. However, due to the rarity of this population, these preliminary results are informative and can help guide future research by better describing the functional processes during social motion perception and similar processes that are impaired in ASD.

## Conclusions

In this study, we demonstrated distinct neural social indices for genetic etiologies of ASD, providing critical insight into the underlying mechanisms of ASD pathology. A unique mechanism was identified for children with ASD and genetic etiology associated with early embryonic development, based upon level of mu attenuation related to social discrimination and patterns over time (i.e., habituation). Our findings implicate genetic heterogeneity as a possible reason for divergent findings in the literature and distinguish the manner by which neural social indices differ between groups and over time. We emphasize the need to continue to discover how phenotypic profiles align within children in specific genotypic subgroups of ASD. Taken together, we predict that future work pursuing phenotypic characterization via the integration of genetic, neural, and behavioral information will continue to inform our understanding of ASD subtypes and will have broad implications for our ability to adopt precision medicine strategies.

## Additional files

Additional file 1: Clinical characterization for children with ASD is provided. Abbreviations: M, male; F, female; LGD, likely gene-disrupting; +, present; -, absent; NC, not completed. (XLS 47 kb)

Additional file 2: Genetic characterization for children with ASD is provided. Bold font highlights likely gene-disrupting mutations (LGD) associated with ASD. Abbreviations: +, present; -, absent; NC, not completed; (M), missense, (N), nonsense; (S), splice; (FS), frameshift; (IV) intronic variant. (XLSX 25 kb)

Additional file 3: Individual slopes from genetics-guided comparisons (LGDM E+ vs LGDM E-) of ongoing dynamic changes of mu attenuation between social and nonsocial motion perception. Power attenuation differential between conditions is averaged within subjects and plotted for LGDM E+ (orange) and LGDM E- (green). Individuals with shared LGDMs (i.e., *CHD8, DYRK1A, SETD2*) are distinguished by "_n". Positive values indicate more mu attenuation for nonsocial relative to social motion perception. Negative values indicate more mu attenuation for social relative to nonsocial motion perception. Left panel: LGDM primarily expressed within early embryonic development (LGDM E+). Right panel: LGDM primarily expressed post-embryonic development (LGDM E-). (TIFF 32871 kb)
