# Supplementary figures and images for: Exploring the heterogeneity of neural social indices for genetically distinct etiologies of autism
Source: J Neurodev Disord. 2017 May 26;9:24. doi: 10.1186/s11689-017-9199-4 (PMC5446693; doi:10.1186/s11689-017-9199-4)

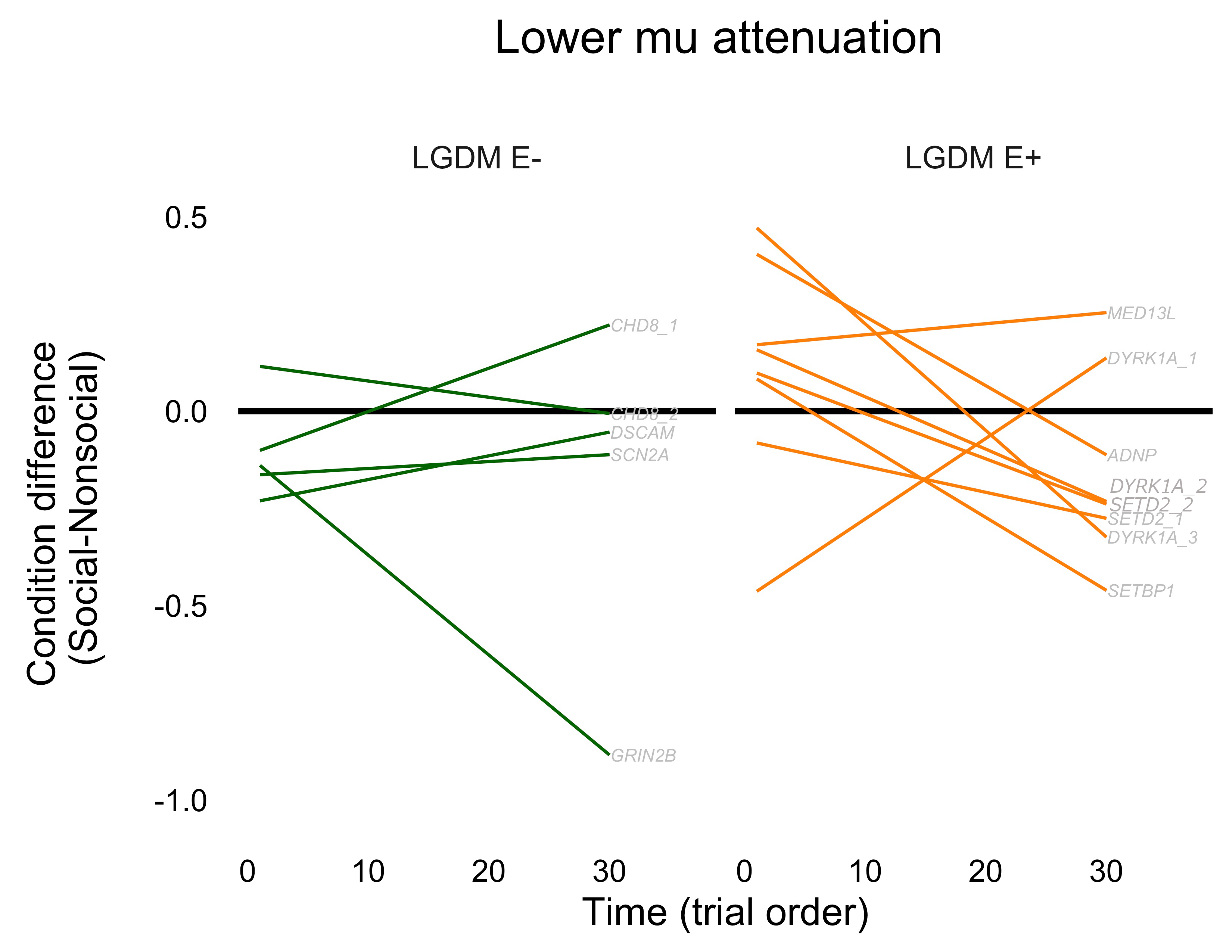

Supplement: Supplementary file 3 — Individual slopes from genetics-guided comparisons (LGDM E+ vs LGDM E-) of ongoing dynamic changes of mu attenuation between social and nonsocial motion perception. Power attenuation differential between conditions is averaged within subjects and plotted for LGDM E+ (orange) and LGDM E- (green). Individuals with shared LGDMs (i.e., CHD8, DYRK1A, SETD2) are distinguished by "_n". Positive values indicate more mu attenuation for nonsocial relative to social motion perception. Negative values indicate more mu attenuation for social relative to nonsocial motion perception. Left panel: LGDM primarily expressed within early embryonic development (LGDM E+). Right panel: LGDM primarily expressed post-embryonic development (LGDM E-). (TIFF 32871 kb) [file 11689_2017_9199_MOESM3_ESM.tiff]
